# N-linked glycosylation of PD-L1/PD-1: an emerging target for cancer diagnosis and treatment

**DOI:** 10.1186/s12967-024-05502-2

**Published:** 2024-07-30

**Authors:** Zhiyun Duan, Runhan Shi, Bo Gao, Jiabin Cai

**Affiliations:** 1 Department of Thoracic Surgery, Zhongshan Hospital, Fudan University, Shanghai, 200032 P.R. China; 2 Department of Immunology, School of Basic Medical Sciences, Shanghai Medical College of Fudan University, Shanghai, 200032 P.R. China; 3 Department of Ophthalmology and Vision Science, Shanghai Eye Ear Nose and Throat Hospital, Fudan University, Shanghai, 200031 P.R. China; 4 Department of Liver Surgery and Transplantation, Zhongshan Hospital, Key Laboratory of Carcinogenesis and Cancer Invasion of Ministry of Education, Liver Cancer Institute, Fudan University, Shanghai, 200032 P.R. China; 5 Department of Liver Surgery, Xiamen Branch, Zhongshan Hospital, Fudan University, Xiamen, 361015 P.R. China

**Keywords:** PD-L1, PD-1, N-linked glycosylation, Immune evasion, Clinical diagnosis, Immunotherapy

## Abstract

During tumorigenesis and progression, the immune checkpoint programmed death-1 (PD-1) and its ligand programmed death ligand-1 (PD-L1) play critical roles in suppressing T cell-mediated anticancer immune responses, leading to T-cell exhaustion and subsequent tumor evasion. Therefore, anti-PD-L1/PD-1 therapy has been an attractive strategy for treating cancer over the past decade. However, the overall efficacy of this approach remains suboptimal, revealing an urgent need for novel insights. Interestingly, increasing evidence indicates that both PD-L1 on tumor cells and PD-1 on tumor-specific T cells undergo extensive N-linked glycosylation, which is essential for the stability and interaction of these proteins, and this modification promotes tumor evasion. In various preclinical models, targeting the N-linked glycosylation of PD-L1/PD-1 was shown to significantly increase the efficacy of PD-L1/PD-1 blockade therapy. Furthermore, deglycosylation of PD-L1 strengthens the signal intensity in PD-L1 immunohistochemistry (IHC) assays, improving the diagnostic and therapeutic relevance of this protein. In this review, we provide an overview of the regulatory mechanisms underlying the N-linked glycosylation of PD-L1/PD-1 as well as the crucial role of N-linked glycosylation in PD-L1/PD-1-mediated immune evasion. In addition, we highlight the promising implications of targeting the N-linked glycosylation of PD-L1/PD-1 in the clinical diagnosis and treatment of cancer. Our review identifies knowledge gaps and sheds new light on the cancer research field.

**Supplementary Information:**

The online version contains supplementary material available at 10.1186/s12967-024-05502-2.

## Introduction

In recent decades, immunotherapy has emerged as an essential tool for treating distinct types of human cancer [1–5]. The advent of immune checkpoint inhibitors, such as antibodies that target cytotoxic T lymphocyte-associated protein 4 (CTLA-4) and programmed death 1/programmed death-ligand 1 (PD-1/PD-L1), has redefined cancer immunotherapy, and these inhibitors have rapidly become an emerging pillar of cancer treatment [6]. Under physiological conditions, the immune system detects and eliminates premalignant or malignant cells in the body; this process is known as immunosurveillance [7]. However, during the development of clinically manifest tumors, a set of cancer cells can acquire the ability to evade the immune response and self-replicate, which eventually leads to tumorigenesis [8]. PD-1, PD-L1 and CTLA-4 are three primary immune checkpoint proteins that can inhibit T-cell function to prevent immune system overactivation. These proteins play critical roles in immune evasion by inhibiting the immune response and allowing cancer cells to evade immune attack [9, 10]. Therefore, antibodies that target immune checkpoints, which are called checkpoint inhibitors, can overcome the inhibitory effects of these proteins on the immune response and activate the immune system to target and destroy cancer cells [11, 12]. Notably, in the context of clinical research, the PD-L1/PD-1 pathway stands out because of the distinct efficacy of targeting this pathway in the treatment of a variety of carcinomas [13, 14]. Since in 2014, when the FDA approved pembrolizumab, the first PD-L1/PD-1 pathway inhibitor, for the treatment of advanced unresectable melanoma [15], multiple clinical trials have indicated that PD-L1/PD-1 pathway inhibitors can induce a potent and durable immune response against cancer cells [16–19]. However, despite the impressive efficacy of anti-PD-L1/PD-1 immunotherapy, a considerable subset of patients demonstrate suboptimal therapeutic responses due to intrinsic and acquired resistance [20, 21]. Hence, more studies are needed to identify therapeutic strategies that are capable of increasing the efficacy of PD-L1/PD-1 blockade immunotherapy.

Initial research on the PD-L1/PD-1 pathway focused on its genetic, transcriptional and posttranscriptional regulation [14, 22, 23]; however, accumulating evidence has revealed that posttranslational modifications (PTMs) of PD-L1/PD-1 are also critical regulators, revealing novel directions for therapeutic approaches that harness the immune system to treat tumors [24–26]. Among the various types of PTMs, glycosylation is one of the most abundant and diverse forms, and it is common in all eukaryotic cells [27, 28]. Interestingly, increasing evidence has shown that both PD-L1 on tumor cells and PD-1 on tumor-specific T cells undergo extensive N-linked glycosylation and that this modification plays a pivotal role in their stability and interaction, ultimately promoting PD-L1/PD-1-mediated immune evasion. In this review, we focus on recent progress in understanding PD-L1/PD-1 N-glycosylation and further highlight the potential therapeutic and diagnostic implications of targeting PD-L1/PD-1 N-linked glycosylation in the context of cancer immunotherapy.

## N-linked glycosylation of PD-L1 and PD-1 and their potential roles in tumorigenesis

### Glycosylation is a fundamental form of posttranslational modification

Glycosylation is mediated by the activity of complex enzymes that attach glycans to proteins or lipids; this modification is mediated by a variety of glycosyltransferases [29, 30]. Glycosylation plays an essential role in a wide range of biological processes, including protein folding, immune regulation, cellular homeostasis, and multiple disease conditions [31]. There are two main types of protein glycosylation in humans: O-linked and N-linked glycosylation [32]. O-linked glycosylation, which involves the attachment of glycans to the oxygen atom (O) of serine (Ser) or threonine (Thr), is a type of glycosylation involved in cell‒cell interactions, signal transduction, virus infection and other biological processes [33, 34]. N-linked glycosylation refers to the attachment of glycans to the nitrogen (N) atom of an asparagine (Asn) residue in a protein, which plays a critical role in protein localization and secretion, immunogenicity and the immune response [35, 36]. As knowledge in the field of glycobiology has grown, recent studies have begun to explore the role of glycosylation in tumorigenesis and its potential implications for diagnosis and therapeutic strategies [28, 37]. Gaining insight into protein glycosylation is critical for revealing the mechanisms underlying protein interactions, cellular signaling, and tumor biology.

### N-linked glycosylation of PD-L1 on tumor cells

**Fig. 1:**
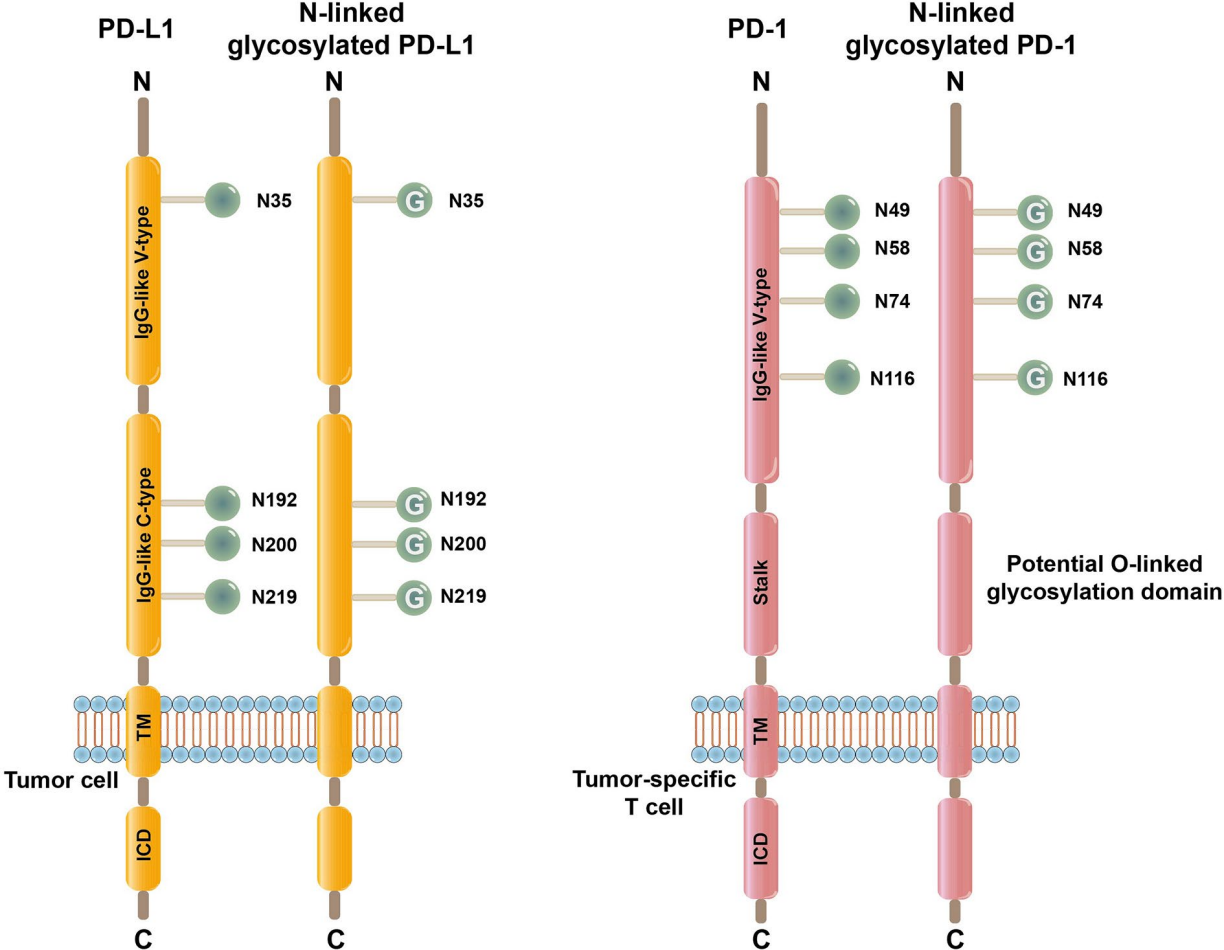
Structures of PD-L1/PD-1 and N-linked glycosylated PD-L1/PD-1. PD-L1, which is a membrane protein that is highly expressed on tumor cells, possesses four asparagine residues that are undergo N-linked glycosylation (N35, N192, N200, and N219) and are distributed across the IgV-like and IgC-like domains of PD-L1. PD-1, which is a membrane protein that is expressed mainly on T cells, possesses four asparagine residues (N49, N58, N74, and N116) that undergo N-linked glycosylation and span the IgV-like domain of PD-1. There are also several potential O-linked glycosylation sites in the stalk domain of PD-1. The numbers represent amino acid residues. TM, transmembrane; IgV, immunoglobulin variable; IgC, immunoglobulin constant

Interestingly, increasing evidence indicates that PD-L1 is heavily glycosylated on various types of tumor cells, including melanoma, breast, lung, and colon cancers. [38–42]According to Western blot profiling, PD-L1 manifests as a range of bands of approximately 55 kDa, whereas naïve PD-L1 is predicted at approximately 33 kDa [43]. After treatment with the recombinant glycosidase peptide-N-glycosidase F (PNGase F), which can efficiently remove N-glycans from the extracellular domain of PD-L1, a single PD-L1 band of the predicted 33 kDa size becomes visible, indicating that PD-L1 mainly undergoes N-linked glycosylation [44]. It has also been reported that treatment with specific N-linked glycosylation inhibitors, rather than O-linked glycosylation inhibitors, changes the electrophoretic pattern of PD-L1, supporting the notion that PD-L1 mainly undergoes N-linked glycosylation [45]. Furthermore, there are four asparagine residues in the PD-L1 extracellular domain, N35, N192, N200, and N219 have been identified as N-glycosylation sites. Mutagenesis of these sites to glutamine can completely abrogate PD-L1 glycosylation [44]. (Fig. 1)

### N-linked glycosylation of PD-1 on tumor-specific T cells

PD-1 has also been reported to undergo extensive N-linked glycosylation on tumor-specific T cells [46, 47]. According to electrophoresis experiments, PD-1 presents two distinct bands of 46 kDa and 32 kDa. Similar to PD-L1, after treatment with PNGase F or N-linked glycosylation inhibitors, a single PD-1 band of ~ 32 kDa becomes visible, indicating that the PD-1 bands with higher molecular weights can be attributed to N-linked glycosylation [46]. There are also four potential N-linked glycosylation sites in the extracellular domain of PD-1, namely, N49, N58, N74, and N116, and mutation of each of these sites leads to a significant reduction in the molecular weight according to SDS‒PAGE electrophoresis [47, 48]. In addition, nonsynonymous single nucleotide polymorphisms (SNPs) at N58 and N116 have been reported in the dbSNP database, which indicates that PD-1 on tumor-specific T cells undergoes a variety of N-glycan modifications [48]. (Fig. 1)

### Aberrant N-glycosylation of PD-L1/PD-1 in tumorigenesis

N-linked glycosylation is reported to play critical roles in tumorigenesis and progression, contributing to abnormal cell matrix interactions, impaired signaling pathways, metastasis, immune evasion, etc. [37, 49–52]. Accumulating evidence indicates that extensive N-linked glycosylation is essential for PD-L1/PD-1-mediated immunosuppression via two mechanism: first, this modification increases protein stability to prevent proteasomal degradation, and second, this modification facilitates the interaction between PD-L1 and PD-1 [44, 45]. N-glycosylation has also been reported to be important for the cell surface localization of these proteins. Furthermore, N-glycans in the PD-1 extracellular domain have been shown to facilitate the binding of some monoclonal antibodies [48, 53, 54]. Collectively, these findings suggest that the N-linked glycosylation of PD-L1/PD-1 plays a crucial role in tumorigenesis mediated by these proteins and that profiling this sophisticated process may shed new light on cancer treatment, which is currently limited.

## Regulatory mechanisms underlying PD-L1/PD-1 N-linked glycosylation and its ability to mediate immune evasion

**Fig. 2:**
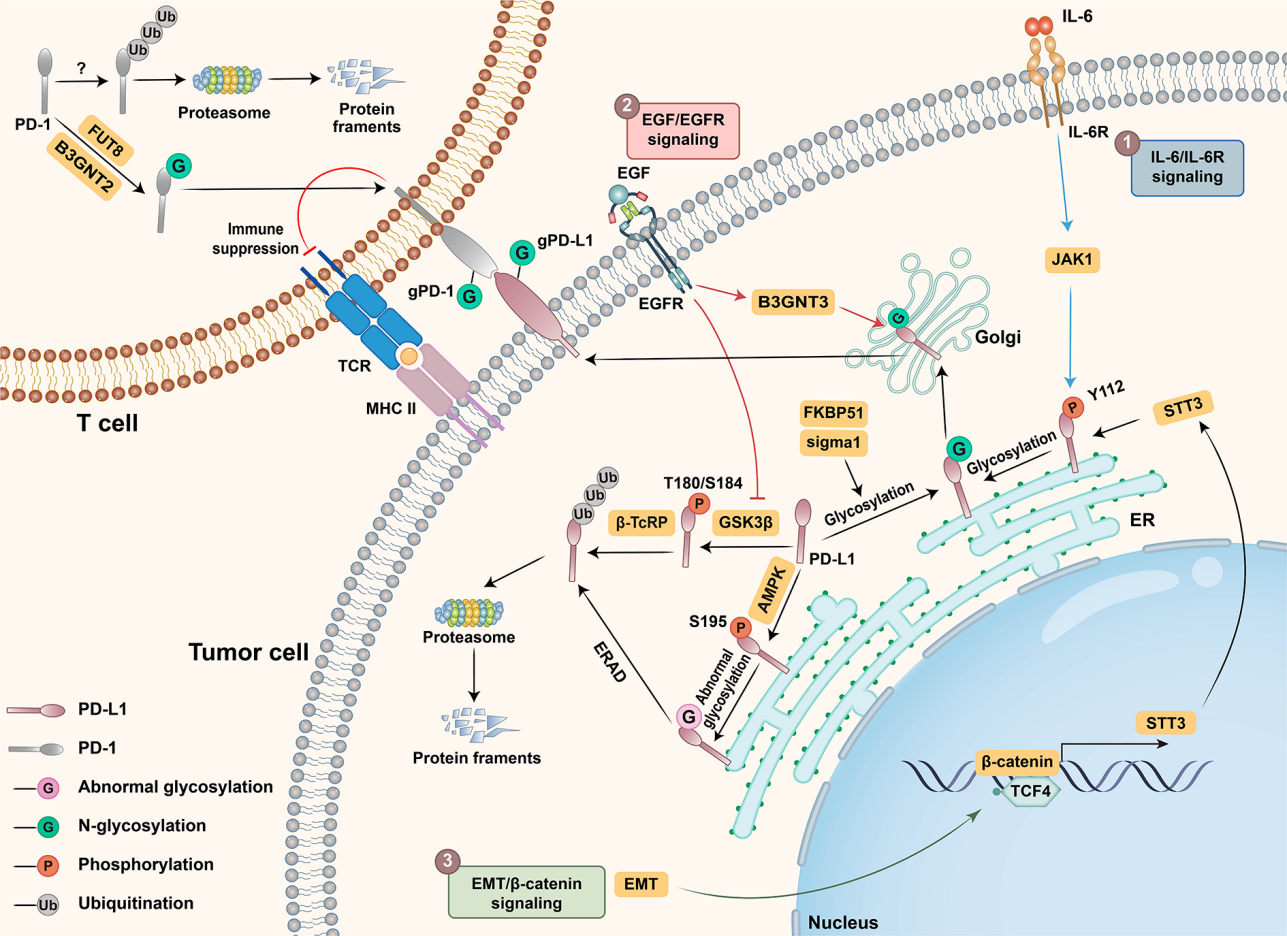
A diagram of the regulatory mechanisms of PD-L1/PD-1 N-linked glycosylation. In tumor cells, GSK3β binds to nonglycosylated PD-L1 and phosphorylates ngPD-L1 at T180/S184, which results in the polyubiquitination of ngPD-L1 via β-TcRP and its 26 S proteasomal degradation. Activated AMPK directly phosphorylates PD-L1 at S195, inducing the abnormal glycosylation of PD-L1 and ERAD. Several signaling pathways are involved in promoting PD-L1 N-glycosylation during tumorigenesis: (1) IL-6/IL-6R signaling activation leads to PD-L1 phosphorylation at Y112 via JAK1 in the ER lumen, which recruits STT3 to N-glycosylate PD-L1; (2) EGF/EGFR signaling inhibits PD-L1 degradation via GSK3β inactivation, while the EGF/EGFR axis induces PD-L1 N-glycosylation by upregulating the glycosyltransferase B3GNT3; and (3) EMT transcriptionally induces STT3 through β-catenin/TCF4, which is recruited to PD-L1 and catalyzes PD-L1 N-glycosylation. In T cells, the N-linked glycosylation of PD-1 via B3GNT2 and FUT8 inhibits PD-1 proteasomal degradation and promotes PD-1 cell-surface localization and PD-1/PD-L1 interaction; FKBP51 and sigma1 function as PD-L1 molecular chaperones, facilitating PD-L1 folding and N-linked glycosylation

N-linked glycosylation is a complex process that is precisely regulated. Since both PD-L1 on tumor cells and PD-1 on tumor-specific T cells undergo extensive N-linked glycosylation in their extracellular domains, an increasing number of studies have focused on elucidating the regulatory mechanisms of PD-L1/PD-1 N-linked glycosylation and its ability to mediate immune evasion. In this section, we focus on several essential signaling pathways that are involved in PD-L1/PD-1 N-linked glycosylation, which are also classical pathways that are involved in cancer development and progression (Fig. 2).

### IL-6/IL-6R axis

Interleukin-6 (IL-6), a critical cytokine that performs various biological functions in immunity, tissue regeneration and metabolism, can bind to its membrane-localized receptor IL-6R and initiate downstream signaling [55]. Dysregulation of IL-6R signaling is known to contribute to inflammatory and lymphoproliferative disorders, such as rheumatoid arthritis and Castleman disease [56–59]. Recently, Chan et al. reported that the IL-6/IL-6R axis is involved in PD-L1 N-linked glycosylation. They reported that the activation of IL-6/IL-6R signaling in cancer cells induces the phosphorylation of PD-L1 at Tyr112 by tyrosine-protein kinase 1 (JAK1). Phosphorylated PD-L1 then recruits the N-glycosyltransferase STT3A to catalyze the N-linked glycosylation of PD-L1 and maintain its stability [60].

### EGF/EGFR axis

Evidence has shown that EGFR signaling activation can increase PD-L1 expression and promote PD-L1/PD-1-mediated immune evasion in EGFR-driven cancer [61, 62]. EGF/EGFR signaling can upregulate PD-L1 transcription through multiple pathways [14], and emerging studies have suggested that EGF-induced posttranslational modifications, particularly N-linked glycosylation, are involved in regulating PD-L1 stability and the PD-L1/PD-1 interaction [44, 45, 63]. Specifically, EGF can induce PD-L1 N-linked glycosylation in basal-like breast cancer, leading to the inhibition of PD-L1 polyubiquitination and subsequent proteasomal degradation by antagonizing the binding of GSK3β [44]. Additionally, EGF/EGFR signaling activation enhances the PD-L1/PD-1 interaction through the upregulation of a glycotransferase, B3GNT3, which is recruited to PD-L1 and mediates PD-L1 N-glycosylation [45].

### EMT/β-catenin/STT3 axis

A recent study revealed that elevated PD-L1 expression in cancer stem cells (CSCs) contributes to immune evasion [64]. Mechanistically, the process of epithelial‒mesenchymal transition (EMT) is involved in the upregulation of PD-L1 [65]. Initially, EMT induces the nuclear translocation of β-catenin, activating the promoters of STT3 isoform genes with the help of another transcription factor, TCF4. STT3 subsequently mediates the N-linked glycosylation of PD-L1 and increases its stability. Additionally, Fat Atypical Cadherin 4 (FAT4), a type of cadherin-associated protein, can interfere with the nuclear translocation of β-catenin and further downregulate STT3A mRNA expression to inhibit PD-L1 N-glycosylation, thus destabilizing PD-L1 and ultimately leading to polyubiquitination-dependent degradation [66].

### FUT-8 and PD-1 N-linked glycosylation

Increasing evidence suggests that PD-1 on tumor-specific T cells also undergoes extensive N-linked glycosylation, which is critical for its cell-surface localization and interaction with PD-L1 [46, 67, 68]. Interestingly, a study revealed that all four N-glycosylation sites, especially N49 and N74, undergo extensive core fucosylation [67], which is a type of glycosylation that has been reported to be essential for proper protein expression and/or ligand‒receptor interactions [69, 70]. Furthermore, this process is catalyzed by FUT8; knocking out FUT8 via the CRISPR-Cas9 system or pharmacological inhibition reduces cell-surface PD-1 expression and enhances T-cell activation [67]. Mechanistically, loss of core fucosylation leads to increased PD-1 polyubiquitination and subsequent proteasome-mediated degradation [68]. FUT8 is significantly upregulated in various types of cancer, and blocking core fucosylation enhances the antitumor immune response in vivo, suggesting that PD-1 expression on tumor-specific T cells is significantly affected by core fucosylated N-glycans [67, 71].

### Others

Considering the crucial role of PD-L1/PD-1 N-linked glycosylation in tumor development and progression, an increasing number of studies have focused on the N-linked glycosylation of PD-L1/PD-1 and the underlying mechanisms involved. For example, it was reported that SEC61G, which is an essential subunit of the Sect. 61 translocon complex, can facilitate the trafficking of newly synthesized PD-L1 to the endoplasmic reticulum (ER) and promote its N-glycosylation, stabilization, and membrane localization, thereby facilitating the immune evasion of EGFR-amplified glioblastoma [72]. Moreover, interferon-stimulated gene 15 (ISG15) can inhibit N-glycosylation of PD-L1 by promoting PD-L1 ubiquitination and degradation [73]. GFAT1, which produces a precursor for N-glycosylation, was shown to be required for PD-L1 expression and stability. Inhibiting GFAT1 suppresses the N-linked glycosylation of PD-L1 and accelerates its proteasomal degradation [74]. GLTD1, an enzyme that transfers glycans to proteins, was reported to stabilize PD-L1 via N-linked glycosylation [75]. In addition, evidence suggests that transmembrane and ubiquitin-like domain-containing protein 1 (TMUB1) can increase PD-L1 N-linked glycosylation and stability by recruiting STT3A to promote PD-L1 maturation [76]. Sigma1 and FKBP151s, which serve as chaperon molecules for PD-L1, are implicated in PD-L1 stabilization in tumor cells by facilitating its folding in the ER and promoting its N-linked glycosylation [77, 78]. Recently, a newly identified glycosyltransferase of PD-L1, namely, B4GALT1, has been shown to directly mediate the N-linked glycosylation of PD-L1, leading to the inhibition of its ubiquitination and proteasome degradation [79, 80]. Together, these findings indicate that N-linked glycosylation of PD-L1 and PD-1 is precisely regulated by multiple signaling pathways and molecules. Leveraging these pathways and molecules may shed new light on cancer diagnosis and treatment, which is reviewed below.

## Deglycosylation of PD-L1 contributes to its detection and prediction of anti-PD-L1/PD-1 immunotherapy outcomes

**Fig. 3:**
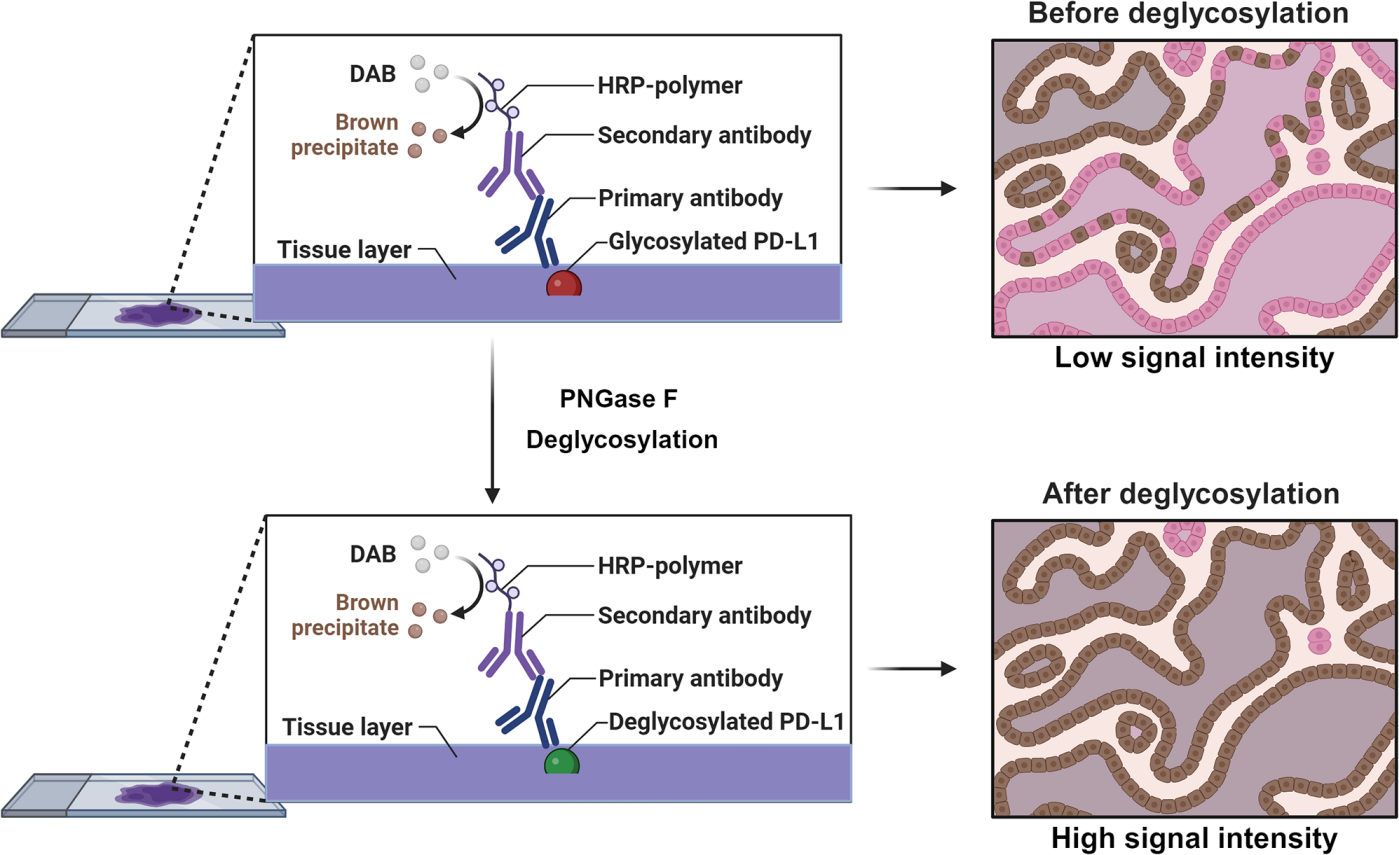
Comparison of heavily N-linked glycosylated PD-L1 and deglycosylated PD-L1 via an IHC assay. N-linked glycans on PD-L1 hinder its recognition by currently common anti-PD-L1 antibodies, leading to suboptimal precision of IHC assays. To address this issue, fixed FFPE tissue slides can be pretreated with the recombinant glycosidase PNGase F to remove N-glycans from PD-L1. This process makes epitopes more accessible for antibody binding, thereby increasing PD-L1 signal intensity and improving PD-L1 detection and therapeutic relevance

Since the response rate to anti-PD-L1/PD-1 immunotherapy remains suboptimal [81], identifying patients who may benefit from PD-L1/PD-1 inhibitor therapy through the use of reliable predictive biomarkers is needed to achieve personalized treatment. Recent evidence has suggested that PD-L1 expression is a promising predictor for stratifying patients for PD-L1/PD-1 inhibitor therapy [82, 83]. However, in many trials, paradoxically, many patients exhibit favorable responses regardless of their PD-L1 expression level in their tumor samples [84–86], making it imperative to improve PD-L1 detection and predictive accuracy. In this section, we summarize new insights into the effects of N-linked glycosylation on diagnosis and how deglycosylation techniques can increase the intensity of PD-L1 in detection assays (Fig. 3).

### N-linked glycosylation inhibits PD-L1 detection

As mentioned above, PD-L1 is a glycoprotein that undergoes extensive N-linked glycosylation in its extracellular domain [45]. N-linked glycans on PD-L1 hinder the recognition and subsequent binding of diagnostic molecules, such as mAbs [87]. However, most commercial PD-L1 antibodies are currently produced without considering the impact of posttranslational modifications, including N-glycosylation, on antigen epitopes [88–90]. Thus, the precision of antibody-based assays remains suboptimal. To address this issue, Lee et al. [87] developed an approach to remove N-linked glycosylation from PD-L1 via the recombinant glycosidase PNGase F. These authors showed that pretreatment with PNGaseF efficiently removes N-glycans from the extracellular domain of PD-L1, and deglycosylation significantly increases the interaction between the antibody and PD-L1. In addition, it has been reported that in lung cancer, the removal of N-glycans from PD-L1 significantly increases the detection efficiency of commercially available PD-L1 antibodies, such as 28 − 8, CAL10, and SP142 [91]. Together, these findings suggest that N-linked glycosylation can inhibit the precise detection of PD-L1 and that deglycosylation of PD-L1 is a promising method for the development of reliable detection assays.

### Deglycosylated PD-L1 is a robust biomarker for predicting anti-PD-L1/PD-1 immunotherapy outcomes

The discrepancy between the expression levels of PD-L1 and the therapeutic outcomes of ICBs has long confused oncologists. Recently, the discovery of PD-L1 N-linked glycosylation and its impact on PD-L1 detection has provided new insight into this inconsistency. A retrospective study demonstrated that deglycosylation of PD-L1 can improve the correlation between PD-L1 expression levels and both overall survival (OS) and disease-free survival (DFS) in patients receiving anti-PD-L1/PD-1 immunotherapy [87]. Furthermore, since deglycosylation can increase the detection accuracy, as shown by increased PD-L1 immunohistochemical (IHC) readouts, this approach will render a subset of patients, who would otherwise be classified as PD-L1-negative and deemed ineligible for ICB therapy, as being eligible for this treatment [87]. Removing N-glycans from PD-L1 makes PD-L1 expression a more reliable biomarker for patient classification and predicting patient response [87]. Taken together, these findings indicate that N-linked glycosylation of PD-L1 partly explains the inconsistency between PD-L1 expression levels and ICB treatment responses and that deglycosylated PD-L1 has the potential to expand the pool of patients who are considered eligible for anti-PD-L1/PD-1 therapy and is a more reliable biomarker for predicting therapeutic outcomes.

## Targeting N-linked glycosylation of PD-L1/PD-1 is an emerging therapeutic strategy to increase the efficacy of cancer immunotherapy

Since the N-linked glycosylation of PD-L1 and PD-1 has been shown to be essential for their ability to mediate immune evasion, scientists have rationally developed multiple small-molecule drugs that target N-glycosylation for use as cancer treatment and evaluated them in several tumor models (Table 1). Furthermore, potential combination strategies to elicit synergistic antitumor effects have also been explored in a set of preclinical trials (Table 2).

**Table 1:** Drugs targeting PD-L1/PD-1 N-linked glycosylation

Drug	Mechanisms	Cancer types	Reference
Metformin	AMPK, activated by metformin, phosphorylates PD-L1 at S195 to induce abnormal glycosylation and ERAD	Breast cancer, Lung cancer, Oral cancer, Endometrial cancer;	[92, 93–95]
D-mannose	Similar to metformin	Breast cancer	[96]
Resveratrol	Blocking α-glucosidase/α-mannosidase and thereby ER retention of abnormally glycosylated form of PD-L1	Breast cancer	[97]
2-DG	As glucose analog interfering with PD-L1 glycosylation processes	Breast cancer, Lung cancer	[98–100]
Etoposide	EMT/β-catenin/STT3 signaling pathway	Breast cancer, Colon cancer	[62]
Niclosamide	1. Inhibiting HuR cytoplasmic translocation, which directly bound to and stabilize PD-L1 mRNA	Breast cancer	[101]
	2. Disturbing glycosylation of PD-L1		
STM108	Antibody directly targeting glycosylated PD-L1	Breast cancer	[41]
ADC	\	Breast cancer	[41, 102, 103]

Abbreviations: ERAD, endoplasmic reticulum-associated degradation; ADC, antibody-drug conjugate

**Table 2:** Combinational strategies based on N-linked glycosylation of PD-L1/PD-1

Combinational strategies	Cancer types	Reference
Metformin + TMV vaccine	Breast cancer, oral cancer	[104]
Metformin + anti-CTLA4	Breast cancer	[92]
D-mannose + anti-PD-1	Breast cancer	[96]
PDG-NVs	Lung cancer	[100]
2-DG + PARPi	Breast cancer	[98]
2-DG + anti-4-1BB	Breast cancer	[99]
Gefitinib + anti-4-1BB	Breast cancer	[99]
Etoposide + anti-Tim-3	Breast cancer, colon cancer	[62]
Niclosamide + anti-PD-1	Breast cancer, lung cancer	[101]
STM108 + MMAE	Breast cancer	[41]
Swainsonine + anti-PD-L1	Lung cancer, melanoma	[105]

Abbreviations: TMV, tumor membrane vesicles; PDG-NVs, nanovesicles with PD-1 displayed on membrane and 2-DG packaged in vesicles; MMAE, anti-mitotic drug monomethyl auristatin E.

### Metformin

Metformin is a well-established medicine that is mainly used to treat type 2 diabetes [92]. However, an increasing number of studies have indicated that metformin may also have promising antitumor properties [104]. A case‒control study showed that metformin treatment can reduce the incidence of various cancer types among patients with type 2 diabetes [106]. Moreover, recent studies revealed that metformin can maintain high cytotoxic T lymphocyte (CTL) activity in tumor tissues [96, 107–109]. Potentially, metformin decreases the stability and membrane localization of PD-L1 by disrupting N-linked glycosylation [97]. Metformin activates AMP-activated protein kinase (AMPK) and directly phosphorylates PD-L1 at S195, resulting in abnormal N-linked glycosylation of PD-L1, causing its retention in the endoplasmic reticulum (ER) and subsequent ER-associated protein degradation (ERAD) [97]. On the basis of these findings, metformin was combined with anti-CTLA4 therapy in a 4T1 breast tumor model, and this approach resulted in significant improvements in tumor burden, survival rate, and CTL activity [97]. It has also been reported that metformin combined with vaccine immunotherapy potently increases the antitumor response via a tumor-intrinsic mechanism and enhances the function of tumor-infiltrated CD8^+^ T cells in several tumor models [98].

### D-mannose

D-mannose serves as the primary monosaccharide component of N-glycans [99]. High levels of D-mannose were shown to inhibit cell growth and enhance sensitivity to major forms of chemotherapy in several types of tumors; this finding indicates that D-mannose is a promising small-molecule drug for cancer treatment [100]. Recently, Zhang et al. [102] reported that D-mannose can restore T-cell function, increasing the sensitivity of tumor cells and tumor-bearing mice to immunotherapy and radiotherapy. Similar to metformin, D-mannose promotes PD-L1 degradation via AMPK activation and the AMPK-mediated phosphorylation of PD-L1 at S195A, which results in impaired N-glycosylation and enhanced polyubiquitination. In vivo, combining D-mannose with anti-PD-1 significantly inhibits tumor growth in 4T1 breast tumor models and prolongs the lifespan of tumor-bearing mice [102].

### Resveratrol

Resveratrol is a common type of dietary polyphenol that is well-known to play a key role in glucose metabolism [103, 101]. Recently, research has shown that when cancer cells are pretreated with resveratrol, the activity of cytotoxic T cells is significantly increased. Similar to metformin and D-mannose, resveratrol can also induce aberrant N-glycosylation of PD-L1, leading to the accumulation of an abnormally N-linked glycosylated form of PD-L1 in the ER and promoting ERAD. In addition, resveratrol can bind to the intracellular domain of PD-L1 and induce its dimerization, which interferes with the PD-L1/PD-1 interaction [93].

### 2-Deoxyglucose (2-DG)

2-DG, which is a glucose analog, was reported to reduce cell-surface PD-L1 expression by disrupting PD-L1 N-linked glycosylation. On this basis, a set of combination strategies have been explored in multiple tumor-bearing murine models. Shao et al. [94] reported that 2-DG can reverse the PARPi-induced upregulation of PD-L1 by deglycosylating PD-L1 in TNBC; thus, the combination of PARP inhibition with 2-DG has more potent antitumor activity. It has also been reported that 2-DG combined with the EGFR inhibitor gefitinib can inhibit PD-L1 N-linked glycosylation and PD-L1-mediated immunosuppression. In TNBC synergistic murine models, combination treatment with 2-DG/gefitinib and the 4-1BB antibody has been shown to enhance antitumor immunity [95]. In the context of chimeric antigen receptor (CAR)-T cell therapy, the utilization of 2-DG reduces the capacity of PD-L1^+^ T3M-4 cells to bind to recombinant human PD-1, allowing CAR-T cells to circumvent immune checkpoint inhibition and increasing their efficacy [40]. In addition, Li et al. [105] developed a genetically engineered PD-1-displaying nanovesicle (P-NV) and reported that loading P-NVs with 2-DG enhances antitumor activity. Together, these findings identify 2-DG as a promising small-molecule drug for combination with current immunotherapies for treating carcinomas.

### gPD-L1 antibodies and ADCs

As mentioned above, N-linked glycans in the extracellular domain of PD-L1 hinder the recognition and binding of diagnostic and therapeutic molecules; thus, the development of monoclonal antibodies (mAbs) that specifically target N-linked glycosylated PD-L1 (gPD-L1) or antibody‒drug conjugates (ADCs) with deglycosylation capabilities has become a promising new therapeutic strategy. Xiao et al. [110] designed an antibody‒enzyme conjugate that can selectively remove sialic acids from the surface of tumor cells, resulting in increased tumor cell killing capacity compared with that of the antibody alone. Another antibody and antimitotic drug conjugate (STM108 + MMAE) was also reported to exert a potent cell-killing effect, while a bystander effect killed adjacent cancer cells that lacked PD-L1 expression [45]. STM108 is an antibody specifically designed to recognize highly N-linked glycosylated PD-L1. In a TNBC murine model, STM108 can effectively inhibit the PD-L1/PD-1 interaction and promote cell-surface PD-L1 internalization and degradation [45]. Furthermore, a tumor microenvironment-activated nanoassembly involving the coassembly of PD-L1- or CTLA-4-antagonizing aptamers and a glucose transporter one inhibitor was shown to significantly decrease PD-L1 N-linked glycosylation. In vivo, the nanoassembly can effectively inhibit N-glycosylation-driven immunosuppression and promote a response to immune checkpoint blockade therapy [111]. Collectively, these findings suggest that targeting gPD-L1 and generating antibody‒drug conjugates are promising directions for cancer treatment in the future.

### gPD-1 antibodies

Similar to PD-L1, multiple N-glycans in the extracellular domain of PD-1 may play crucial roles in the binding of anti-PD-1 antibodies. Compared with current FDA-approved anti-PD-1 antibodies, mAbs that specifically target glycosylated PD-1 exhibit higher binding affinity for PD-1, effectively hindering the PD-L1/PD-1 interaction and leading to potent antitumor immunity [46]. In addition, PD-1 N58 glycosylation can promote the binding of some monoclonal antibodies, including cemiplimab [53], camrelizumab [48], and MW11-h317 [54]. Together, these findings suggest that targeting PD-1 N-glycosylation is also a promising strategy for improving the efficacy of immune therapy.

### Others

Etoposide is a common medication that has been used to treat various cancers. A recent study suggested that it can disrupt N-glycosylation by inhibiting the EMT/β-catenin/STT3/PD-L1 axis, leading to the downregulation of PD-L1 and the sensitization of cancer cells to anti-Tim-3 therapy by altering PD-L1 N-linked glycosylation [65]. An inhibitor of α-mannosidase, namely, swainsonine, was reported to interrupt PD-L1 N-glycosylation, and the combination of swainsonine and anti-PD-L1 exerted a synergistic therapeutic effect on lung cancer and melanoma [112]. It was also demonstrated that niclosamid can enhance CTL activity by disrupting PD-1 N-linked glycosylation and significantly improve the efficacy of anti-PD-1 immunotherapy in vivo. [113]. As mentioned above, the IL-6/JAK1 pathway can induce PD-L1 phosphorylation at Y112 to promote PD-L1 N-glycosylation. The combination of anti-IL-6 and anti-Tim-3 has been shown to be an effective targeted therapeutic strategy [60].

## Perspective and conclusion

Immune evasion is a hallmark of cancer, and it allows tumors to resist the host immune system and escape immune detection and destruction. Here, we reviewed the N-linked glycosylated modification of PD-L1/PD-1 and its critical role in PD-L1/PD-1-mediated immune evasion, which consequently contributes to tumorigenesis. We also explored the potential implications of the N-linked glycosylation of PD-L1/PD-1 in the clinical diagnosis and treatment of cancer, suggesting that targeting N-linked glycosylation might be a promising strategy for more precise diagnosis and more efficient immunotherapy. Notably, although substantial strides have been made in understanding PD-L1/PD-1 glycosylation and its role in immune evasion, the mechanisms underlying this complex process are not fully understood; for example, the alterations in PD-L1/PD-1 N-linked glycosylation that are involved in the development of cancer are not well understood. In addition, research on the role of PD-L1/PD-1 N-linked glycosylation in diagnosis and treatment is currently in the preclinical stage. Whether patients in the real world could benefit from these newly proposed promising strategies requires a series of clinical trials with increasing scale. A major challenge is that small-molecule drugs, such as 2-DG and D-mannose, inhibit protein N-linked glycosylation in a general manner. How to precisely target these drugs to tumor cells and reduce systemic side effects is an issue that urgently needs to be solved. To achieve more precise treatment, antibody‒drug conjugates (ADCs) and tumor-targeted nanovesicles, which are emerging strategies for drug delivery, may have broad application prospects in the future. Moreover, in addition to PD-L1 on cancer cells and PD-1 on T cells, N-linked glycans are also commonly found on a variety of cell-surface immune checkpoint proteins, such as PD-L2 [114], B7-H3 [115], B7-H4 [116], and VISTA [117]. It would be interesting to determine whether the presence of N-glycans in the extracellular domains of these proteins plays a role in immune evasion in vivo and hinders their detection in vitro [43]. Research has reported that the inhibition of B7-H4 glycosylation could be favorably combined with current therapeutic strategies to achieve a superior response rate in immunologically cold breast cancers [118]. Interestingly, previous studies have focused mainly on the N-glycosylation of PD-L1/PD-1, but a recent study revealed that O-linked N-acetylglucosamine (O-GlcNAcylation) can promote tumor immune evasion by inhibiting PD-L1 lysosomal degradation [119]. Moreover, a previously undescribed site in the stalk region of the PD-1 protein that undergoes O-linked glycosylation was also identified [120]. Thus, further investigations of the O-linked glycosylation of PD-L1/PD-1 and its role in clinical diagnosis and treatment would be worthwhile.

In conclusion, the elucidation of PD-L1/PD-1 glycosylation has shed new light on the clinical diagnosis and treatment of cancer. Moreover, further research on the underlying mechanisms and the implications of this process for the real world is needed. With the development of glycobiology, harnessing the glycosylation of immune checkpoint points, such as PD-L1 and PD-1, would be a promising strategy to benefit patients in the future.

### Electronic supplementary material

Below is the link to the electronic supplementary material.

Supplementary Material 1

## Data Availability

Not applicable.

## Abbreviations

PD-1: Programmed cell death protein 1
PD-L1: Programmed cell death 1 ligand 1
CTLA-4: Cytotoxic T lymphocyte-associated antigen-4
ICB: Immune checkpoint blockade
PTMs: Post-translational modifications
PNGase F: Peptide-N-glycosidase F
SNP: Single nucleotide polymorphisms
JAK1: Tyrosine-protein kinase 1
CSCs: Cancer stem cells
EMT: Epithelial-mesenchymal transition
FAT4: Fat Atypical Cadherin 4
ER: Endoplasmic reticulum
ISG15: Interferon-stimulated gene 15
TMUB1: Transmembrane and ubiquitin-like domain-containing protein 1
OS: Overall survival
DFS: Disease-free survival
IHC: Immunohistochemistry
CTL: Cytotoxic T lymphocyte
AMPK: AMP-activated protein kinase
ERAD: ER-associated protein degradation
2-DG: 2-deoxyglucose
PAPRi: Poly(ADP-ribose) polymerase inhibitor
gPD-L1: Glycosylated PD-L1
ADC: Antibody-drug conjugate
MMAE: Anti-mitotic drug monomethyl auristatin E
O-GlcNAcylation: O-linked N-acetylglucosamine
HGS: Hepatocyte growth factor-regulated tyrosine kinase substrate
