# Supplementary material for: N-linked glycosylation of PD-L1/PD-1: an emerging target for cancer diagnosis and treatment
Source: J Transl Med. 2024 Jul 30;22:705. doi: 10.1186/s12967-024-05502-2 (PMC11290144; doi:10.1186/s12967-024-05502-2)
Supplement: Supplementary file 1 — Supplementary Material 1 [file 12967_2024_5502_MOESM1_ESM.docx]

Manuscript ID: JTRM-S-24-06710

**MS TITLE:**

**N-linked Glycosylation of PD-L1/PD-1: An Emerging Target for Cancer Diagnosis and Treatment**

Dear editor:

We are happy to have the opportunity to revise our manuscript titled "N-linked Glycosylation of PD-L1/PD-1: An Emerging Target for Cancer Diagnosis and Treatment". In response to the reviewers’ questions and suggestions, we meticulously revised the manuscript and made corresponding supplementations and corrections point-by-point. We hope the revised manuscript will satisfy the reviewers and meet the quality standard of the journal.

Thanks again for your reconsideration and looking forward to your early reply.

Sincerely Yours,

Jiabin Cai, MD

Department of Liver Surgery and Transplantation,

Liver Cancer Institute,

Zhongshan Hospital, Fudan University,

Shanghai 200032, P.R. China.

E-mail: cai.jiabin@zs-hospital.sh.cn

**Reviewers’ Comments to Authors**

**Reviewer ^#1^ (Comments for the Author):**

It is clear, comprehensive, and suggests that the review could provide valuable insights and drive future research. The focus on both mechanistic and clinical aspects ensures that the review will be relevant to a wide audience, including researchers and clinicians in oncology but a check for language correction is strongly suggested to ensure precision.

**Authors’ Response:**

Thanks for comments. We are delighted to hear that you found our review to be clear, comprehensive, and potentially valuable in providing insights to stimulate future research. It is indeed our intention to cover both the mechanistic and clinical aspects of the topic, ensuring that our work resonates with a broad audience.

Considering your suggestion for language correction, we have thoroughly proofread the manuscript to refine the language and enhance its precision. We also have engaged the services of American Journal Experts (AJE), a leading provider of professional editing services for scientific publications. We hope that these efforts further improve the quality of our work and make it more accessible to potential readers. AJE Certificate of Editing will be uploaded as a supplementary material. Thank you once again for your constructive suggestions.
